# Treatment of osteochondral fracture of lateral femoral condyle after patella dislocation with anchor absorbable sutures: A new surgical technique and a case report

**DOI:** 10.1097/MD.0000000000032104

**Published:** 2022-12-16

**Authors:** Liang Wu, Chao Liu, Bing Jiang, Lijiang He

**Affiliations:** a Department of Orthopedic Surgery, First People’s Hospital of Linpin District, Hangzhou, Zhejiang, China; b Department of General Surgery, Medicine Faculty of Universitas Prima Indonesia, North Sumatra, Indonesia; c Department of General Surgery, Daocheng Country People’s Hospital, Sichuan, China; d Department of Orthopedic Surgery, Second People’s Hospital of Yuhang District, Hangzhou, Zhejiang, China.

**Keywords:** absorbable internal fixation, dislocation of patella, femoral condyle, osteochondral fracture

## Abstract

Osteochondral fracture (OCF) in weight-bearing area of lateral femoral condyle (LFC) is a rare combined injury caused by patellar dislocation.

**Patient concerns:**

A 15-year-old female student accidentally sprained her right knee while participating in sports activities. The patient felt pain in his right knee and limited movement. After hospitalization, the patients underwent computed tomography scan and magnetic resonance examination.

**Diagnosis:**

According to the imaging results, patellar dislocation combined with OCF of LFC was considered in diagnosis.

**Interventions:**

Through the lateral parapatellar approach, we reduced the osteochondral mass and bundled it with absorbable sutures of anchors.

**Outcomes:**

The functional and radiographic outcome were satisfactory at 18 months after operation.

**Lessons:**

Anchor absorbable suture bridge fixation for this kind of OCF is not only effective, but also economical.

## 1. Introduction

Cartilage injury of lateral femoral condyle (LFC) caused by patellar dislocation is very common, with an incidence rate of 31% to 40%.^[1,2]^ However, most LFC cartilage injuries are located in the anterior non-weight-bearing area. However, in recent years, some authors^[3–5]^ reported OCF involving the weight-bearing area of LFC. A meta-analysis by Kühle et al^[6]^ show that there is no unified treatment for osteochondral fractures (OCF) of knee joint at present, and the overall failure rate is 17%. Treatment options include loose body removal, microfracture, multiple internal fixation and so on. We report a case of patellar dislocation with OCF in the weight-bearing area of LFC. We used anchor absorbable suture bridge to fix osteochondral mass, and obtained good functional and imaging results at the final follow-up.

## 2. Case presentation

**Figure 1.**
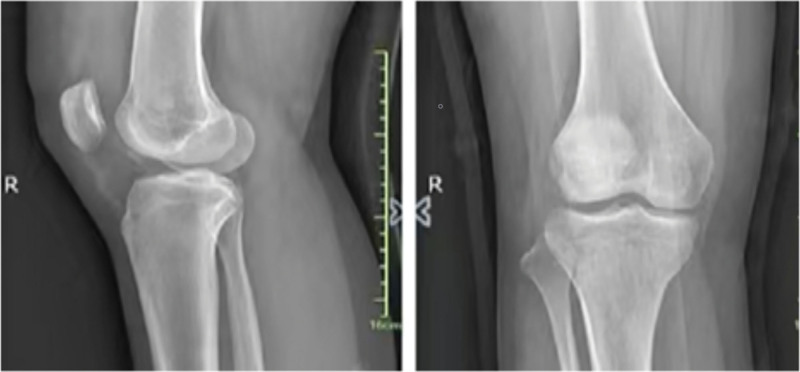
Radiographs of knee joint show loose body in joint.

**Figure 2.**
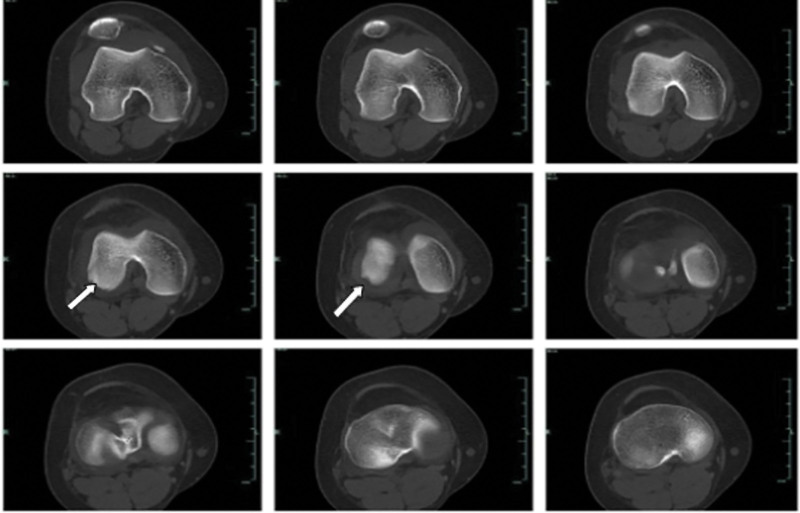
The white arrow indicate the defect area.

**Figure 3.**
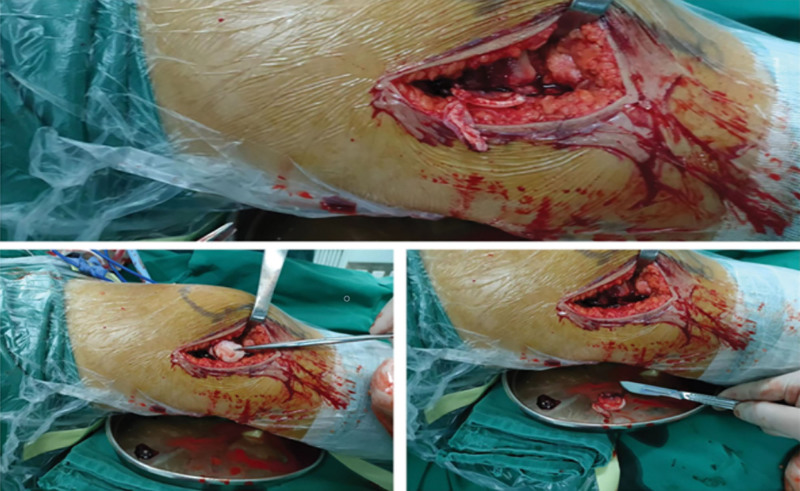
Two cartilage masses can be seen during the operation.

**Figure 4.**
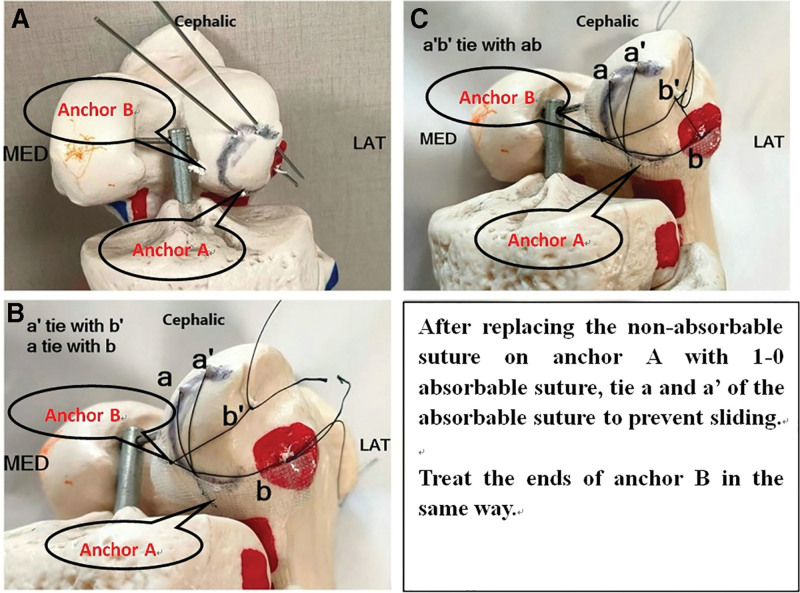
Schematic diagram of surgical process.

**Figure 5.**
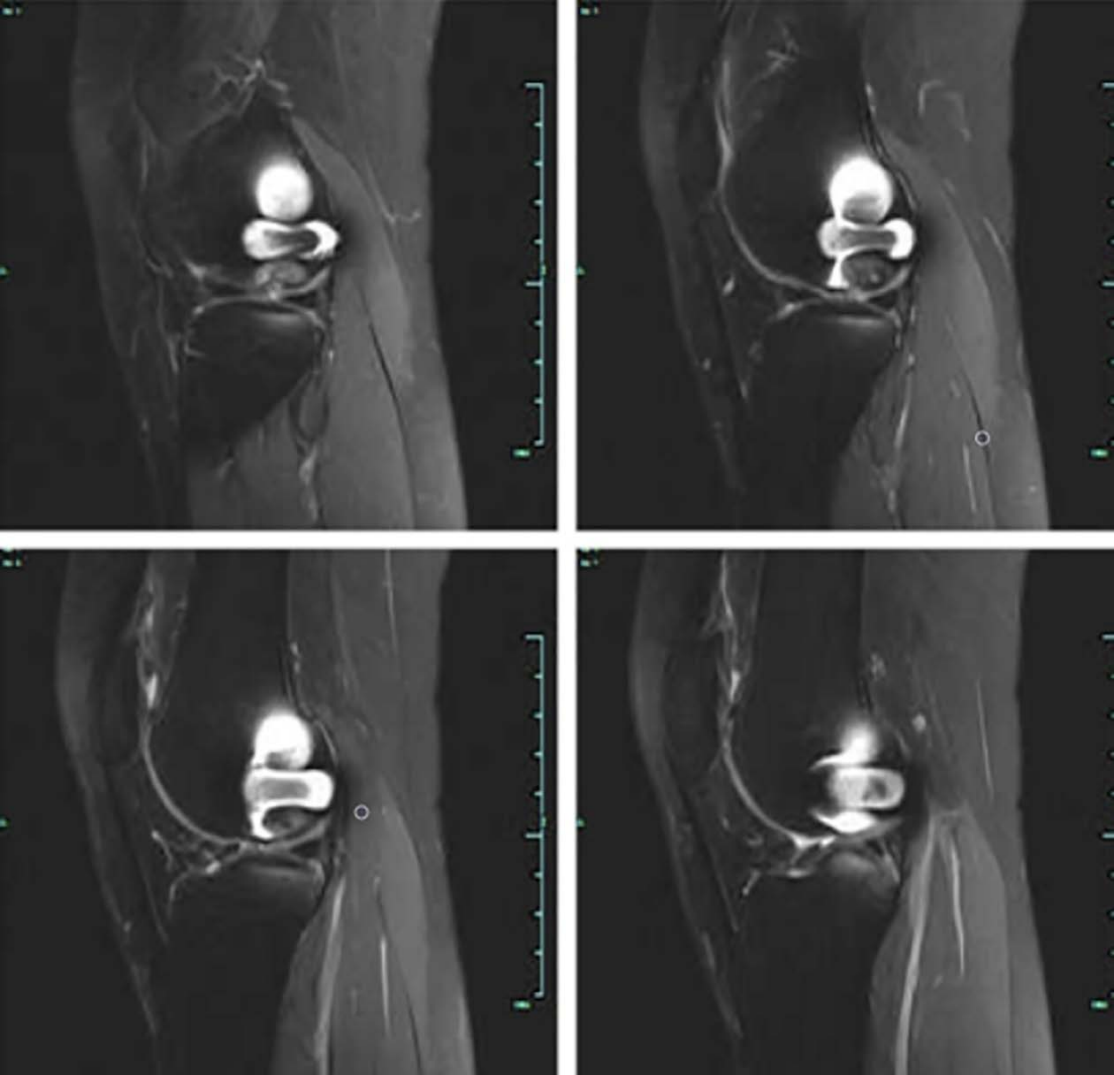
Sagittal MRI images were reexamined 18 months after operation, MRI = magnetic resonance.

**Figure 6.**
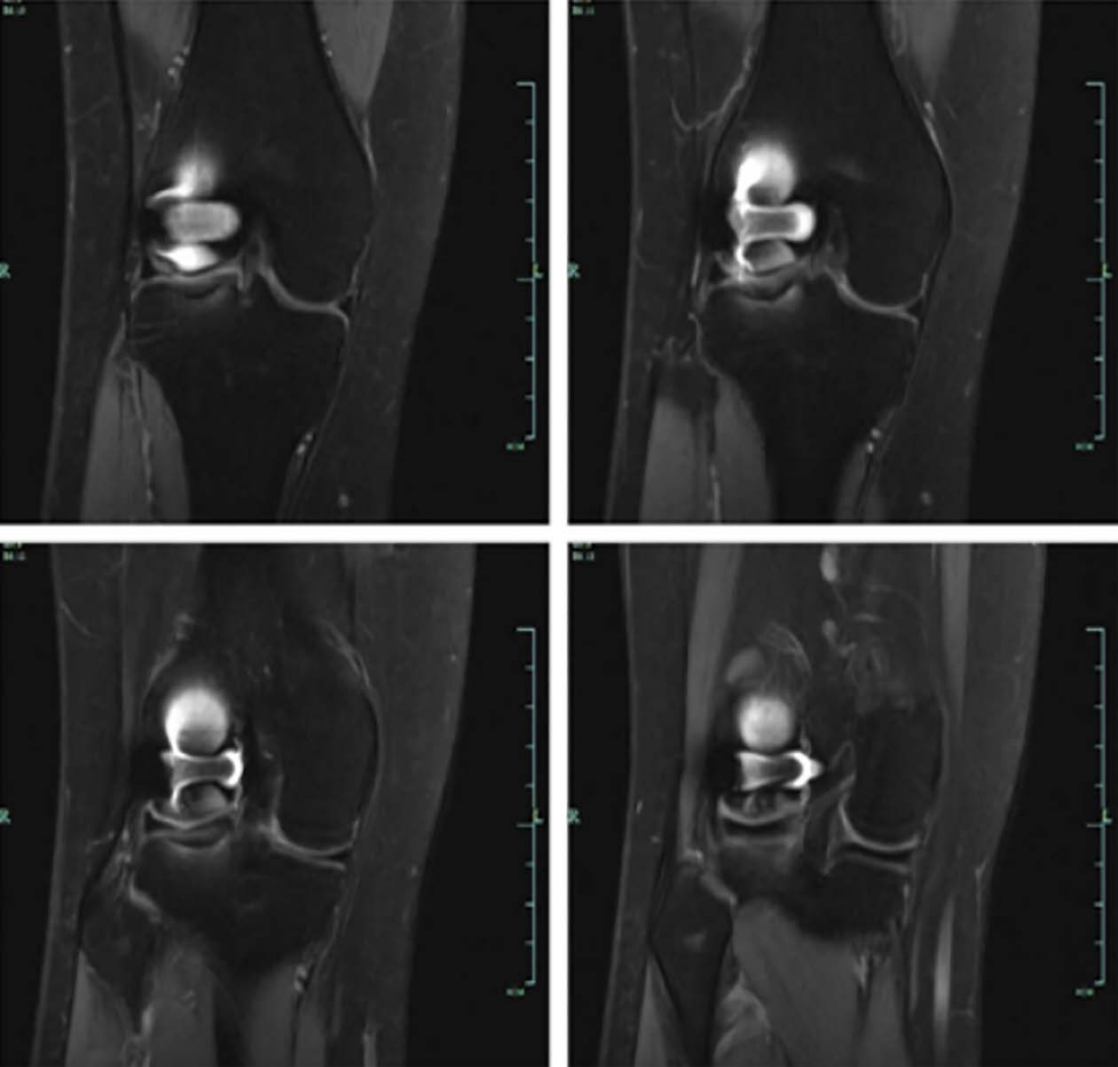
Coronal MRI images were reexamined 18 months after operation, MRI = magnetic resonance.

A patient, 15-year-old, female student. Complained of swelling and pain of the right knee after spraining during sports activities, demonstrated painful limited motion. The patient had no previous history of patella dislocation and pain around the knee. When the patient was sent to the emergency room, the right knee swelled obviously, tenderness over the medial border of the patella, the apprehension test was positive, lateral stress test was negative, and the knee range of motion:F/E 90°/0°. Preliminary X-ray examination showed osteochondral defects of LFC and loose body in knee joint (Fig. 1). computed tomography scan and magnetic resonance (MRI) examination of knee joint further confirmed loose body within the knee joint, osteochondral defect in weight-bearing area of LFC and avulsion of medial patellofemoral ligament (Fig. 2). The patellar height was in the normal range (Caton-Deschamp index 1.0).^[7]^ The development of trochlear sulcus of femur was classified as type A according to Dejour et al,^[8]^ and the TT-TG^[9]^ was 15 mm. Three days after injury, the lateral parapatellar incision of the right knee was performed under general anesthesia, OCF reduction and fixation of the lateral condyle was performed. During the operation, we found that 2.5*2. 0 cm osteochondral mass was stripped from the weight-bearing area of the LFC, 2.0*0. 5 cm cartilage mass was stripped from nonweight-bearing area of the LFC, and no osteochondral mass was found at the medial edge of patella (Fig. 3). During the operation, 2 4.5 mm anchor (Smith @ nephew TIWNFIX Ultra PK Suture Anchor) was inserted into the posterior edge and medial edge of the cartilage mass in the weight-bearing area, and then 2 non-absorbable sutures on each anchor were replaced by an absorbable suture (ETHICON VICRYL PLUS VCP359H), and finally the 2 ends of the absorbable suture were knotted to prevent sliding. Two bone tunnels are made from anteromedial to posterolateral with 2 mm Kirschner wire at the front edge of osteochondral mass. After the osteochondral mass was fixed in situ to the lateral condyle of the femur, 2 suture ends of the posterior suture anchor penetrate into the front bone tunnels respectively, and after penetrating from the LFC, they are knotted and fixed with 2 suture ends of medial suture anchor respectively (Fig. 4). After the incision was closed in layers, the lower limb was splinted for 6 weeks, isometric exercises for the quadriceps began the day after surgery. Active and passive knee flexion exercise of the right knee was gradually strengthened 6 weeks after operation. Knee flexion was limited less than 60° within 8 weeks after operation, partial weight-bearing was allowed at 8 weeks, followed by full weight bearing from 12 weeks after operation. Postoperative reexamination of computed tomography scan showed that the bone block was well reduced. After 6 months, the patient could resume normal sporting activities, and the knee joint extension and flexion were normal without knee instability and pain. After physical examination, it was found that apprehension test was negative, patellar glide and tilt tests was negative. MRI reexamination at 18 months after operation showed that the osteochondral mass healed well (Figs. 5 and 6), and the lysholm score was 95 points, which was very good.

## 3. Discussion

Matthewson et al^[10]^ believe OCF in weight-bearing area of LFC with patellar dislocation is caused by the shearing forces between the LFC and the lateral tibial plateau as they pivot under load. Many author think these injuries are caused by the impact between the patella and femoral condyle with a knee flexed over 90°.^[11,12]^ The bone marrow edema at the posterolateral aspect of the LFC suggest that the knee joint is highly flexed during patellar dislocation.

Osteochondral defects of LFC are usually caused by lateral patellar dislocation, most of which are located on the medial side of patella.^[13]^ There are also many reports on OCF in non-weight-bearing area of LFC.^[14,15]^ Diederichs et al^[16]^ suggest that a first patellar dislocation is often treated conservatively, and loose body removal, microfracture and internal fixation should be selected according to the size and location of osteochondral block. Pa š a et al^[17]^ reported that 10 patients with patellar osteochondral mass less than 2.7 mm^2^ caused by patellar dislocation still achieved good function only by taking out the loose body, and no patellar dislocation was found. Hawkins et al^[18]^ found that the recurrent dislocation rate of patients with primary patellar dislocation is related to congenital femoral trochlear dysplasia, high patellar position and large TT-TG. Moreover, even if the medial patellar retinaculum is strengthened, the patient still has symptoms such as anterior knee pain. Shah et al^[19]^ systematically reviewed the recurrent patellar dislocation and found that the complication rate of patellar medial collateral ligament reconstruction was as high as 26.1%. Gang et al^[20]^ found that there was no statistically significant difference between surgical treatment and non-surgical treatment in patients with patellar side injury of medial collateral ligament of patella. This patient has no patella alta, well developed femoral trochlea, no obvious increase of TT-TG and no previous patellar instability. We do not do patellar medial collateral ligament repair to reduce complications such as knee joint adhesion.

The treatment options for OCF of LFC include: loose body removal, microfracture, open reduction and internal fixation, cartilage transplantation, autologous or allogeneic osteochondral transplantation, etc.^[21]^ Matthewson et al^[21]^ reported for the first time that patellar dislocation complicated with OCF of LFC was treated with early internal fixation and external fixation to avoid early weight bearing, and achieved good results. Studies by Gesslein et al^[22]^ show that open reduction and internal fixation of LFC with OCF is better than loose body removal. Mashoof et al^[11]^ reported 7 cases of OCF in the weight-bearing area of LFC caused by patellar dislocation, of which 3 cases were treated with bioabsorbable screw fixation, but the follow-up results were not reported. Callewier et al^[23]^ reported a patient who used absorbable pin fixation to treat OCF in the weight-bearing area of LFC. After 1 year follow-up, good functional and radiographic outcome were obtained. Friederichs et al^[24]^ reported cases of opposing articular surface cartilage injury caused by bioabsorbable screws, which required second operation. Li et al^[25]^ used absorbable suture to treat OCF caused by patellar dislocation and achieved good medium-term results. Zhou et al^[26]^ used suture anchor to treat LFC OCF under arthroscope, and achieved good clinical results. However, some patients had suture removal during the second arthroscopy because of suture irritation. We replaced the anchor suture with (ETHICON VICRYL PLUS VCP 359H) suture during the operation, which is an attempt based on the research of Li,^[25]^ in order to avoid the second operation.

## 4. Conclusions

Patellar dislocation with OCF in weight-bearing area of LFC is a rare injury in adolescents. When patients have tenderness along the medial edge of patella and knee joint effusion, it is necessary to actively improve MRI examination, to rule out osteochondral injury. When the patient has patellar dislocation with OCF in the weight-bearing area of LFC, surgical treatment and internal fixation is the treatment of choice if the OCF can be fixed. Anchor absorbable suture bridge fixation is rigid enough, which can avoid second operation, save cost and good outcome could be expected, which is worth exploring; Of course, a large number of clinical data are needed for further comparative study.

## Author contributions

**Data curation:** Liang Wu, Bing Jiang.

**Writing – original draft:** Lijiang He.

**Writing – review & editing:** Chao Liu.
